# An audit of fresh frozen plasma usage and effect of fresh frozen plasma on the pre–transfusion international normalized ratio

**DOI:** 10.4103/0973-6247.67024

**Published:** 2010-07

**Authors:** S. A. Shinagare, N. N. Angarkar, S. R. Desai, M. R. Naniwadekar

**Affiliations:** *Department of Pathology, Krishna Institute of Medical Sciences, Karad, Maharashtra, India*

**Keywords:** Audit, effect on INR, fresh frozen plasma

## Abstract

**Objective:**

To audit the usage of fresh frozen plasma (FFP) and to study the effect of FFP on the pre-transfusion international normalized ratio (INR).

**Materials and Methods:**

Medical records of 100 consecutive patients who received FFP in our institute were retrospectively studied. FFP usage was classified as appropriate or inappropriate based on the guidelines by the National Health and Medical Research Council and The Australasian Society for Blood Transfusion. Pre-and post-transfusion INR were recorded and the effect of FFP on the pre-transfusion INR was studied in patients who appropriately received FFP. Relationship between the pre-transfusion INR and improvement in the INR per unit of FFP was studied using Pearson’s correlation.

**Results:**

Total 325 units were issued for the 100 patients (37 males and 63 females, mean age 33 years, range 1-65 years). Obstetrics and gynecology and medicine departments requested most units of FFP. Total 197 units (60.6%) in 67 patients were appropriately transfused and 128 units (39.4%) in 33 patients were inappropriately used. Mean improvement in the pre-transfusion INR per unit of FFP was 0.79 (median 0.53, range 0-3.5, SD 0.94). A significant improvement in the pre-transfusion INR per unit of FFP was seen in 64.9% patients. A linear relationship was noted between the pre-transfusion INR and improvement in INR per unit of FFP (r=0.89, degree of freedom 55).

**Conclusion:**

Proportion of inappropriate FFP usage remains high. A significant improvement in INR is more likely with a high pre-transfusion INR. The improvement in INR per unit of FFP is also more with higher pre-transfusion INR.

## Introduction

Fresh frozen plasma (FFP) is a blood product extracted from plasma and frozen to –30°C or below within 6 h after collection.[1] It contains plasma proteins and all the coagulation factors, including the labile factors V and VIII.[2] The use of FFP has significantly increased in the past 10 years and its usage continues to increase. There are certain situations where FFP is clearly indicated, such as in patients with coagulopathy resulting from DIC who are undergoing invasive procedures or having active bleed, in patients with liver failure with active bleed and in patients with thrombotic thrombocytopenic purpura (TTP).[3] However, there are many instances, such as specific factor deficiency, patients needing volume expansion, where FFP use is either controversial or not indicated.[4] Furthermore, FFP contains antibodies capable of causing complications like hemolytic reactions and transfusion-related acute lung injury. It is also capable of transmitting viruses like human immunodeficiency virus, hepatitis B virus, and C virus.[5] Hence the use of FFP is not without potential danger and it should be used only if clearly indicated. The National Health and Medical Research Council and the Australasian Society for Blood Transfusion (NHMRC/ASBT guidelines),[6] the College of American Pathologists,[1] and the British Committee for Standards in Haematology[7] have published guidelines to highlight these issues and to minimize the misuse of FFP. However, many studies from around the world still report a high frequency of inappropriate FFP usage.[1,8–21]

Literature search revealed 15 audits of FFP usage all over the world,[1,8–21] which included only two audits from India, by Kakkar et al,[13] and by Chaudhary *et al*.[19] Our institute is a tertiary care teaching hospital in rural part of India. Consistent with the global trend, FFP usage has been increasing in our institute since past few years. Hence we took up a study on the institute’s FFP usage with the specific aims of auditing our FFP usage according to the age of the patient, indications for FFP administration, appropriate and inappropriate usage of FFP and, the improvement in INR with FFP. We also studied the amount of FFP administered and improvement in INR in each case, thus studying the actual effect of FFP on coagulation profile. This aspect of FFP usage has not been adequately studied so far. We believe this may help in anticipating the dose of FFP required to improve the coagulation variables and may also help us in setting up policies to improve the utility and reduce the wastage of this important blood product.

## Material and Methods

Medical records of 100 consecutive patients who received FFP in our hospital from May 2006 to April 2007 were retrospectively studied. The following data were collected: provisional clinical diagnosis, indication for FFP, specialty of the requesting clinician, the demographic data including age and gender of the patient, number of units transfused and, patient’s pre and post-transfusion international normalized ratio (INR). The usage of FFP was divided into two categories, appropriate and inappropriate, based on the guidelines published by the National Health and Medical Research Council and The Australasian Society for Blood Transfusion (NHMRC/ASBT guidelines)[6] [Table 1]. Infusion of 10-15 ml/kg body weight of the patient was considered adequate dose.[22] FFP usage was called appropriate if it was according to NHMRC/ASBT guidelines and was administered in appropriate dosage. Patients were screened for other co-morbid factors, such as history of bleeding disorders, vitamin K deficiency, and hematological malignancies. The appropriateness of FFP transfusion in such patients was decided based on the final clinical indication for requesting the FFP and whether it was in accordance with the NHMRC/ASBT guidelines.

**Table 1:** National Health And Medical Research Council and the Australasian Society for Blood Transfusion fresh frozen plasma, 2002

Appropriate if any one of the following applicable, likely to be inappropriate if none applicable
INR or APTT high and liver disease before major surgery or invasive procedure
INR or APTT high and liver failure
INR or APTT high and acute disseminated intravascular coagulation
INR or APTT high and excessive bleeding
INR or APTT high before an invasive procedure
INR or APTT high before, during or after major surgery
INR high and warfarin effect present and massive blood loss or emergency surgery
Correction of single factor deficiency when a specific factor was not available
Treatment of thrombotic thrombocytopenic purpura

The PT was assayed and INR calculated on a single test coagulometer (Model 501, Dutch Diagnostics, Zutphen, The Netherlands). The pre- and post-transfusion INRs were calculated in order to study the effect of FFP on coagulation variables. The post-transfusion PT was done within 1 hour of completion of transfusion. Due to short time gap between measurements of coagulation parameters, the total variation was assumed to be equal to the actual FFP-induced variation with no contribution from biologic variation. The improvement in the INR per unit of FFP was calculated. Pre-tranfusion INR was correlated with the improvement in INR per unit of FFP, using Pearson’s linear correlation. We also calculated the magnitude of improvement in INR compared to the pre-transfusion INR, per unit of FFP. The number of patients who showed a significant improvement in the INR was determined using the following formula derived by Holland and Brooks.[23]

Significant Change ≥ 8.9%[pre-transfusion INR]

According to this formula, a decrease of 8.9% or more in a pre-transfusion INR per unit of FFP was considered as a significant improvement.

## Results

A total of 325 units of fresh frozen plasma were issued for the 100 patients in our study group, which included 37 (37%) males and 63 (63%) females with a mean age of 33 years (range 1-65 years). FFP was most commonly transfused in the patient age group of 16 to 30 years. Obstetrics and gynecology department requested most units of FFP (40 patients, 115 units), followed by the medicine (25 patients, 81 units) and surgery (19 patients, 74 units) departments [Table 2]. The most common indications for FFP usage were acute disseminated intravascular coagulation (DIC) with high INR (38 patients, 120 units), followed by excessive bleeding with high INR (18 patients, 47 units) and volume depletion (16 patients, 65 units) [Table 3]. The usage of FFP was categorized as appropriate if it was transfused in adequate dosage (10-15 ml/kg body weight) for indication as per the NHMRC and ASBT guidelines.[6] If FFP was transfused in an inadequate dosage or for un-indicated reason as per the guidelines, we labeled it as inappropriate use. We concluded that FFP was transfused appropriately in 67 patients amounting to 197 units (60.6%). 33 patients received inappropriate transfusion of FFP, amounting to 128 units (39.4%). Acute DIC with high INR was the most common indication for appropriate FFP transfusion, while the use of FFP for volume expansion was the most frequent cause of inappropriate FFP usage.

**Table 2:** Faculty-wise distribution

Faculty	Number of Patients	Number of FFP units
Obstetrics and Gynecology	40	115 (35.4%)
Medicine	25	81 (24.9%)
Surgery	19	74 (22.8%)
Pediatrics	14	46 (14.2%)
Radiotherapy	2	9 (2.8%)
Total	100	325

**Table 3:** Indications for fresh frozen plasma

Indications for fresh frozen plasma	Total number of patients	Units
Acute DIC with high INR	38	120 (36.9%)
Excessive bleeding with high INR	18	47 (14.5%)
Liver failure with high INR	11	33 (10.2%)
Liver disease without high INR	5	10 (3.1%)
Sepsis	9	41 (12.6%)
Severe iron deficiency anemia	3	09 (2.8%)
For volume expansion	16	65 (20.0%)
Total	100	325

**Figure 1:**
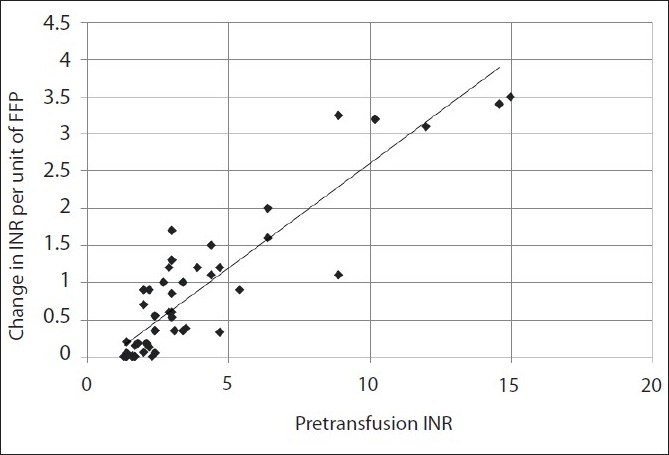
Linear relationship between the pre-transfusion INR and improvement in INR per unit of FFP (Pearson linear correlation, r=0.89, degree of freedom 55)

**Figure 2:**
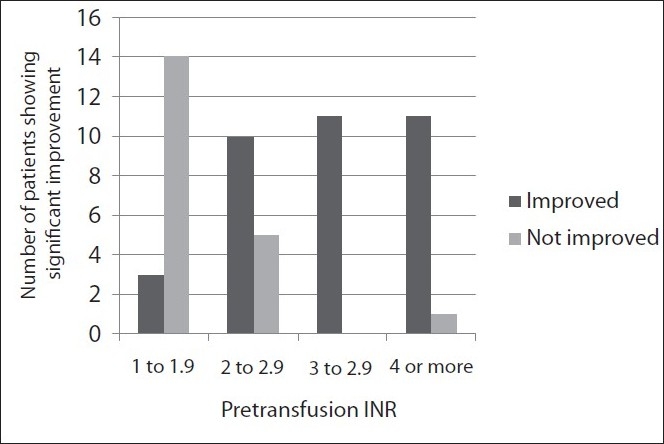
Graph showing pretransfusion INR and number of patients showing significant improvement per unit of FFP

Out of the 67 patients who appropriately received FFP transfusion, 55 patients received only FFP and 12 patients received FFP along with other supplements such as whole blood, PCV or vitamin K. In order to study the effect of FFP transfusion on INR, patients who received other supplements were excluded, except for two patients who received vitamin K along with FFP. Vitamin K requires 4 to 8 h to exert its effect, and the post-transfusion PT/INR assay was done within 1 hour. Therefore, these two patients were included. Thus, pre- and post-transfusion INRs were compared in a total of 57 patients. In this group of patients, the mean pre-transfusion INR was 3.6 (median 2.4, range 1.3 to 14.6, Standard deviation 3.10) and mean post-transfusion INR was 1.8 (median 1.5, range 0.8 to 7, SD 0.95). In this same patient group, the improvement in INR per unit of FFP was calculated. The mean improvement in the pre-transfusion INR per unit of FFP was 0.79 (median 0.53, range 0 to 3.5, SD 0.94). A linear relationship was noted between the pre-transfusion INR and improvement in INR per unit of FFP [Figure 1]. Pearson’s correlation coefficient (r) was 0.89 (degree of freedom 55, critical value for significance 0.273), showing that the net improvement in INR was more with higher pre-transfusion INR. Using the formula derived by Holland and Brooks,[23] a change of 8.9% or more in the pre-transfusion INR per unit of FFP was considered as a significant change. Out of 57 patients, 37 patients (64.9%) showed a significant improvement in the pre-transfusion INR and 20 patients (35.1%) showed no significant benefit. We found that with a low pre-transfusion INR, the proportion of patients showing significant improvement in INR was low, while most of the patients with a higher pre-transfusion INR showed a significant improvement in INR [Figure 2].

## Discussion

While the use of fresh frozen plasma (FFP) is increasing globally, it must be remembered that it is associated with potential risks to the recipient. Many studies have shown a high incidence of inappropriate use of FFP.[1,8–21] Inappropriate use not only leads to a wastage of limited resources depriving more needy patients, but also leads to an increased healthcare cost and increased risk of transfusion related complications. Therefore, there is a need for more prudent use of this expensive blood product. Various guidelines for appropriate FFP use have been proposed.[6,7,22–32] However many of these are mainly expert consensus rather than recommendations derived from meticulous scientific studies. Moreover, the threshold of PT and aPTT prolongation of > 1-1.5 times normal was based on outdated retrospective studies, and PT and aPTT themselves are poor predictors of perioperative bleeding especially in patients with a negative bleeding history. Therefore the utility of routine preoperative coagulation testing has been questioned.[32,33] According to British Committee for Standards in Haematology guidelines,[32] bleeding history including family history, details of prior surgeries, and anticoagulant treatment should be taken prior to surgery. Patients with a negative bleeding history do not require routine preoperative coagulation testing. However, some recent papers still recommend routinely performing PT, aPTT, and platelet count prior to surgery and invasive procedures in adults and children.[34] Use of FFP in patients with excessive warfarin effect also needs further clarification. According to NHMRC/ASBT guidelines,[6] FFP transfusion is considered appropriate in patients with excessive warfarin effect only if they have a massive bleeding or have emergency surgery. It is known that FFP is not the optimal treatment for excessive warfarin effect and should never be used for the reversal of warfarin anticoagulation in the absence of severe bleeding.[7] Prothrombin complex concentrate (PCC) is effective in reversing the effect of warfarin, and FFP may be used when there is severe bleeding and PCC is not available.

In our study, FFP was most often used in patients of age range 16-30 years. In our institute, the obstetrics and gynecology department requested the most units of FFP. Acute DIC with a high INR and excessive bleeding with a high INR, including cases of antepartum and post partum hemorrhage were the most common indications for FFP. Our study showed inappropriate FFP transfusion in 33% patients, amounting to 128 units (39.4%). While the exact figures differ, various published audits also show a high proportion of inappropriate FFP usage.[1,8–21] In our study, the use of FFP for volume expansion was the most frequent form of FFP misuse. We believe that the widespread uncertainty about the appropriate indications of FFP among the clinicians is the cause of this high rate of unindicated FFP transfusions. In our experience, we found two common reasons behind the inappropriate FFP requests. Some clinicians were not aware of the guidelines, while some clinicians tend to use FFP as a “precaution” against litigations and disputes. This warrants efforts to raise awareness among clinicians, that appropriate FFP transfusion requires presence of active bleeding or an invasive procedure in a setting of coagulopathy and prolongation of PT or aPTT. For instance, in our study FFP was transfused in 16 patients for volume expansion. In these cases, other alternatives like plasma expanders should have been used instead of FFP.

As mentioned earlier, the effect of FFP on the pre-transfusion INR was considered in a group of 57 patients who received appropriate transfusion of FFP. In this group, 64.9% patients showed a significant improvement in the INR per unit of FFP, using the formula derived by Holland and Brooks.[23] We studied two effects of FFP on INR, first, the objective improvement in the pre-transfusion INR value and second, the probability of a significant improvement in the pre-transfusion INR. There was a linear relationship (r=0.89, degree of freedom 55, critical value for significance 0.273) between the pre-transfusion INR and improvement in INR value per unit of FFP. This is consistent with the findings of Holland and Brooks.[23] In other words, the improvement in INR was more in those patients who had a high pre-transfusion INR. We also found that significant improvement in the INR was less likely in patients who had a low pre-transfusion INR and more likely in those patients who had a high pre-transfusion INR.

To summarize, those with a high pre-transfusion INR are more likely to be benefited with FFP and show more improvement in INR per unit of FFP. These observations may help us refine the institutional policies regarding FFP usage. Since FFP is a precious product, we propose a preferential use of FFP for those patients who fulfill the guidelines and have a high pre-transfusion INR. We feel that further studies on these lines are required in order to improve the utilization of this important blood product.
